# BaCarb™: anovel bioinorganic matrix for local drug delivery

**DOI:** 10.1186/1753-6561-8-S4-P77

**Published:** 2014-10-01

**Authors:** Karina Cesca, Maximiliano L Cacicedo, Valéria E Bossio, Guillermo R Castro, Luismar M Porto

**Affiliations:** 1 Chemical and Food Engineering Department - UFSC, Florianópolis-SC, Brazil; 2 Nanobiomaterials Laboratory - UNLP, La Plata, Argentina

## Background

Treatment of localized tumor by chemotherapy is mostly used as an adjuvant to surgery to protect against or delay the progression of disseminated metastatic diseases or for treatment when other local therapies, such as surgery are not feasible. Local drug delivery offers high potential as a therapeutic choice against premature oncological stages or with isolated cancers since it is more effective in reducing cancer recurrences and avoiding the undesirable side effects of drug spreading along the body [1]. Several applications of the bacterial cellulose in the biomedical field were proposed, like wound healing membranes based on the similarities to the human skin, artificial blood vessels, and as replacement of cornea, valve prosthesis, urethra, cartilage, etc. Also, the use of cellulose membranes has been postulated for drug delivery of hormones and proteins and it was recently fully reviewed [2,3]. Doxorubicin hydrochloride (dox) is commonly used in the treatment of many solid tumors, such as breast, lung, stomach or ovarian cancer and sarcoma [4]. In the present work, we develop a bacterial cellulose membrane for encapsulation of Doxorubicin and containing λ-Carr and CaCO_3 _as a potential implant device for local treatment of solid tumors. The *in vitro *Dox release kinetics from the matrices was studied at different pH values and also in different matrices.

## Methods

The system of bacterial cellulose containing hybrid microparticles (HMPs) was carried out by colloidal crystallization in the presence of CaCl_2_, glycine (Gly), Na_2_CO_3 _and λ-carrageenan (Carr) [5]. To evaluate the potential for incorporation of chemotherapy, the hybrid system was immersed in a doxorubicin solution (Dox). The *in vitro *kinetics was carried out in PBS buffer.

## Results end conclusions

The developed systems have the advantages of being synthesized on bacterialcellulose, a biomaterial broadly used in many biomedicalengineering applications, where cellulose is used as a physical support and a biological scaffold for tissue formation. Addition of Carr to the matrix raises the Dox loading only to 16.6%. However, the presence of CaCO_3 _raises up to 83.3% and 87.3% the Dox loaded into BC and BC-Carr, respectively. The presence of HMPs attached to MC allowed the increase of the Dox loading and made strict control of the drug release produced by local changes of pH made by tumoral cells. The Dox administration can be modulated by the amount of Dox required in the treatment and also by the amount of CaCO_3 _and their crystallization conditions.

The system has the simplicity of Dox encapsulation in the HMPs by a technical procedure that allows to establish the amount of Dox/matrix required for specific treatment. The high loading capability of the Dox-CaCO_3_-Carr-BC matrix is advantageous since it is reducing the drug waste. Finally, the biodegradable matrix could be used for the treatment of oncological pathologies, particularly for breast cancer by local delivery of doxorubicin and defining the amount of drug to be released.
